# LPS-Toll-Like Receptor-Mediated Signaling on Expression of Protein S and C4b-Binding Protein in the Liver

**DOI:** 10.1155/2010/189561

**Published:** 2010-08-18

**Authors:** Tatsuya Hayashi, Koji Suzuki

**Affiliations:** ^1^Department of Molecular Pathobiology, Mie University Graduate School of Medicine, Tsu-city, Mie 514-8507, Japan; ^2^Department of Biochemistry, Mie Prefectural College of Nursing, Tsu-city, Mie 514-0116, Japan

## Abstract

Protein S (PS), mainly synthesized in hepatocytes and endothelial cells, plays a critical role as a cofactor of anticoagulant activated protein C (APC). PS activity is regulated by C4b-binding protein (C4BP), structurally composed of seven *α*-chains (C4BP*α*) and a *β*-chain (C4BP*β*). In this paper, based primarily on our previous studies, we review the lipopolysaccharide (LPS)-induced signaling which affects expression of PS and C4BP in the liver. Our *in vivo* studies in rats showed that after LPS injection, plasma PS levels are significantly decreased, whereas plasma C4BP levels first are transiently decreased after 2 to 12 hours and then significantly increased after 24 hours. LPS decreases PS antigen and mRNA levels in both hepatocytes and sinusoidal endothelial cells (SECs), and decreases C4BP antigen and both C4BP*α* and C4BP*β* mRNA levels in hepatocytes. Antirat CD14 and antirat Toll-like receptor (TLR)-4 antibodies inhibited LPS-induced NF*κ*B activation in both hepatocytes and SECs. Furthermore, inhibitors of NF*κ*B and MEK recovered the LPS-induced decreased expression of PS in both cell types and the LPS-induced decreased expression of C4BP in hepatocytes. These data suggest that the LPS-induced decrease in PS expression in hepatocytes and SECs and LPS-induced decrease in C4BP expression in hepatocytes are mediated by MEK/ERK signaling and NF*κ*B activation and that membrane-bound CD14 and TLR-4 are involved in this mechanism.

## 1. Introduction

**Figure 1:**
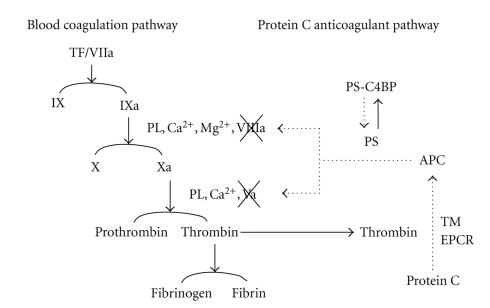
*Blood coagulation pathway and protein C anticoagulant pathway*. Thrombin, bound to thrombomodulin (TM), activates protein C which binds to endothelial protein C receptor (EPCR). Activated protein C (APC) inactivates coagulation factor Va (Va) and factor VIIIa (VIIIa) in the presence of protein S (PS). PS circulates in free form and in complex with C4b-binding protein (C4BP) in plasma, and the free form of PS plays a role as a cofactor of APC. TF: Tissue factor, PL: Phospholipids.

**Figure 2:**
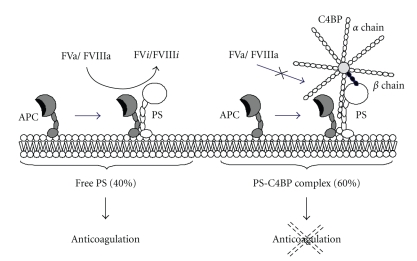
*Functions of PS and PS-C4BP complex*. PS circulates in free form (about 40%) and in complex form with C4BP (about 60%) in plasma. C4BP is comprised of seven *α* chains and a *β* chain, and PS binds to the *β* chain. APC proteolytically converts factors Va and VIIIa (Va/VIIIa) into inactivated factors Va and VIIIa (V*i*/VIII*i*) in the presence of free form PS, but not the complex form PS with C4BP.

Protein S (PS), a vitamin K-dependent plasma glycoprotein (*M*r 75,000), is a physiologically important regulator of blood coagulation, as patients with hereditary PS deficiency have severe thrombotic diseases [1–3]. In the blood coagulation system, PS is a cofactor of activated protein C (APC), which inactivates the blood coagulation factor Va and factor VIIIa [4–6] (Figure 1). PS may also directly inhibit the prothrombinase complex by binding to factor Va and factor Xa [7, 8]. In human plasma, PS circulates in free form and in complex with C4b-binding protein (C4BP), a protein of the classical complement pathway [9, 10]. C4BP consists of seven *α* chains (*M*r 70,000) and a *β* chain (*M*r 45,000), with the *β* chain being important in complex formation with PS [11–13]. Both forms of PS are capable of binding to APC, but only the free form acts as a cofactor [14, 15] (Figure 2). Additionally, PS may promote phagocytosis of apoptotic cells by macrophages [16] and mediate neuroprotection via APC [17]. These observations suggest that PS has important functions in both blood coagulation and inflammation. 

 Human PS is mainly synthesized in hepatocytes, endothelial cells and megakaryocytes [18–20], and C4BP in hepatocytes [21]. Acquired decreased plasma PS levels have been associated with liver diseases [22], pregnancy [23], oral contraceptive use [24], sepsis-associated disseminated intravascular coagulation (DIC) [25], and systemic lupus erythematosus [26], and patients with these conditions have an increased incidence of thrombotic events. Decreased levels of free PS may also lead to thrombotic tendency; therefore, it is believed that increased levels of plasma C4BP, as found in type III PS deficiency, are a risk factor of thrombosis [27].

Previously, we demonstrated that PS acts as a cofactor for APC in rats, and that it forms a complex with rat C4BP, as in humans [28]. Studies in various species demonstrated that both humans and rats have the PS-C4BP complex in plasma [10, 14, 28, 29]; therefore, rats are considered to be the most appropriate animal to study the pathophysiological role of PS. In this paper, we describe changes in the plasma levels of PS and C4BP. We also evaluate the *in vitro* effect of LPS on PS and C4BP expression in hepatocytes and/or sinusoidal endothelial cells (SECs) isolated from rats and the LPS-mediated signaling that affects PS and C4BP expression in these cells.

## 2. Effect of LPS on PS Expression In Vivo and In Vitro

**Figure 3:**
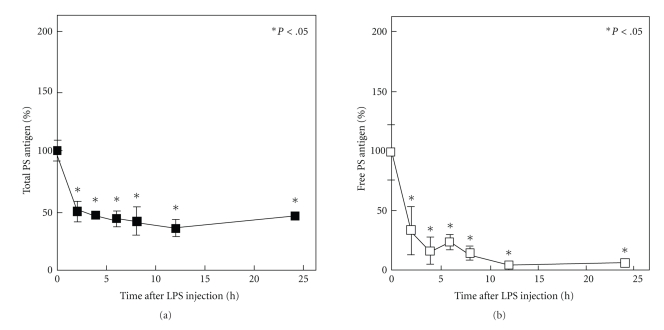
*Changes in plasma levels of total PS and free PS in LPS-treated rats*. Citrated plasma was obtained from three rats treated with LPS intraperitoneally (2 mg · kg^−1^) at each time point to determine (a) plasma total PS antigen levels and (b) plasma free PS antigen levels, as described previously in [31]. Data are expressed as the mean ± S.D. (*n* = 3). *P < .05 versus time 0.

We showed that in a rat endotoxemia model [30, 31], prepared by intraperitoneal injection of LPS, the total PS level in plasma was significantly decreased while the free PS level was markedly decreased in plasma after LPS injection (Figures 3(a) and 3(b)).  APC cofactor activity of plasma isolated from rats 24 hours after LPS injection was also evaluated by activated partial thromboplastin time (APTT), suggesting that plasma obtained from rats 24 hours after LPS injection prolonged the APTT significantly less than plasma from nontreated rats (data not shown). These results suggest that LPS-induced reduction of plasma PS is a cause of thrombotic tendency in patients with sepsis. Thses results were consistent with the report by Hesselvik et al. that patients with sepsis have decreased plasma levels of PS, and that this is associated with thrombotic events  [32]. In the liver, PS mRNA transiently decreased from 4 hours to 8 hours after LPS treatment and then returned to baseline levels; however, the plasma antigen level of PS did not recover concomitantly with the PS mRNA expression [30]. The detailed mechanism of this phenomenon is unclear, thus future investigations are needed. The *in vitro* studies using hepatocytes and SECs isolated from normal rats indicated that LPS dose-dependently decreased mRNA expression of PS in both cells, and these decreases occurred at the transcriptional level [30]. These data suggest that decreased plasma level of PS in LPS-treated rats is mainly due to reduced PS mRNA expression in both hepatocytes and SECs.

## 3. Effect of LPS on C4BP Expression In Vivo and In Vitro

**Figure 4:**
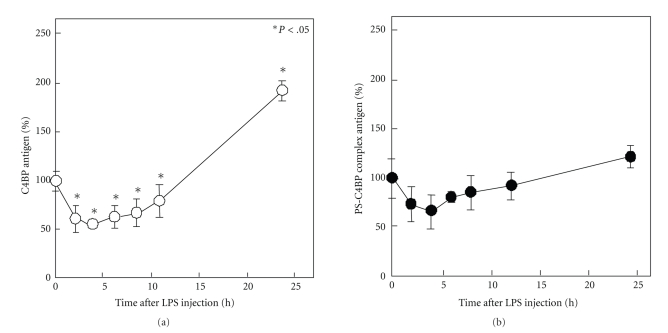
*Changes in plasma levels of C4BP and PS-C4BP complex in LPS-treated rats*. Citrated plasma was obtained from three rats treated with LPS intraperitoneally (2 mg · kg^−1^) at each time point to determine (a) plasma C4BP antigen levels and (b) plasma PS-C4BP complex antigen levels, as described previously in [31]. Data are expressed as the mean ± S.D. (*n* = 3). *P < .05 versus time 0.

It is reported that plasma C4BP levels are significantly increased in patients with severe infection and septic shock [32], but it is unknown whether C4BP expression in the liver is directly affected by LPS. We examined the effect of LPS on C4BP expression *in vivo* in the liver of rats and *in vitro* in isolated rat hepatocytes. We observed that LPS transiently decreased the plasma level of C4BP antigen with a maximum decrease between 4 hours and 6 hours, followed by a significant increase by 24 hours after LPS injection (Figure 4(a)) [31]. This result was consistent with the data that free PS was significantly decreased for 24 hours after LPS injection (Figure 3(b)). However, the PS-C4BP complex level was not significantly changed within 24 hours after LPS injection (Figure 4(b)). The *in vitro* studies using hepatocytes isolated from normal rats indicated that LPS directly decreased both C4BP*α* and C4BP*β* mRNA expression in hepatocytes [31]. These data suggest that the early decrease of plasma C4BP is caused by a direct effect of LPS. It is also reported that interleukin (IL)-6 increased C4BP expression in HepG2 cells [33], suggesting that in LPS-treated rats the relatively late increase in the plasma level of C4BP is caused by IL-6. It is reported that IL-6 also increased PS expression in HepG2 cells [34] and in rat hepatocytes [30]. However, it is unclear whether IL-6 is one of the causes of thrombotic tendency. To clarify this point we prepared IL-6-injected rats, and *in vivo* effect of IL-6 on plasma PS, C4BP and PS-C4BP complex level was examined. Our data indicated that both C4BP and PS-C4BP complex levels in plasma are increased until 8 hours after IL-6 injection, and then gradually decreased, and free PS level is decreased 24 hours after IL-6 injection, so that APC cofactor activity of plasma 24 hours after IL-6 injection is decreased as compared with nontreated rats (data not shown). These results suggest that IL-6 causes thrombotic tendency by increasing C4BP expression in hepatocytes followed by increasing plasma PS-C4BP complex and decreasing plasma free PS level. These results also suggest that IL-6-induced reduction of free plasma PS is also the cause of thrombotic tendency in endotoxemia rats, and the major effect of IL-6 is increasing of C4BP expression in hepatocyte rather than increasing of PS expression in hepatocytes and SECs.

## 4. Signal Transduction Pathway Involved in LPS-Induced Expression of PS in Hepatocytes and SECs and C4BP in Hepatocytes

CD14 and TLR-4 are necessary for signal transduction induced by LPS in which LPS, bound to CD14, can interact with TLR-4 in the presence of myeloid differentiation protein-2 (MD-2) [35, 36]. It is known that induction by LPS on NF*κ*B activation occurs via CD14 and TLR-4 in human endothelial cells [37], and mouse hepatocytes [38]. Recently, we showed that rat hepatocytes and SECs also express CD14 and TLR-4 [30]. Further, gel mobility-shift assay indicated that anti-CD14 and anti-TLR-4 antibodies inhibit LPS-induced NF*κ*B activation in both hepatocytes and SECs. These results suggest that LPS also induced NF*κ*B activation via CD14 and TLR-4 in both rat hepatocytes and SECs [30].

**Figure 5:**
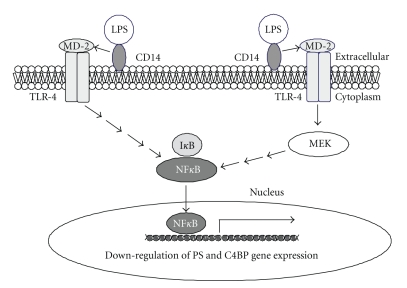
*Mechanism of LPS-induced decreased expression of PS and C4BP in hepatocytes*. LPS binds to membrane-bound CD14 and TLR-4, followed by activation of MEK/ERK and NF*κ*B. Activated NF*κ*B interacts with the promoter regions of the PS and C4BP*α* and C4BP*β* genes, leading to decreased PS expression in hepatocytes and SECs, and decreased C4BP expression in hepatocytes.

 Furthermore, we found that a NF*κ*B inhibitor blocked LPS-induced decreased expression of PS in both hepatocytes and SECs and the LPS-induced decreased C4BP*β* expression in hepatocytes [30, 31], suggesting that NF*κ*B activation is involved in the expression of PS and C4BP in hepatocytes and/or endothelial cells. These data also suggest that the rat PS, C4BP*α* and C4BP*β* gene promoters contain a NF*κ*B consensus sequence. In addition, we found that a MEK inhibitor blocks LPS-induced reduction of PS expression in hepatocytes and SECs, and LPS-induced reduction of C4BP in hepatocytes [30, 31]. These data are consistent with previous studies [36, 37], that LPS induces activation of the MEK/ERK pathway and NF*κ*B nuclear translocation in hepatocytes and endothelial cells. These findings suggest that NF*κ*B activation and MEK/ERK pathway, but not the protein kinase C, JNK and p38 MAPK pathways, are linked to LPS-induced decreased PS expression in rat hepatocytes and SECs, and also linked to LPS-induced decreased C4BP expression in rat hepatocytes (Figure 5).

## 5. Effect of Inflammatory Cytokines on PS and C4BP Expression in Hepatocytes and SECs

LPS stimulates monocytes and endothelial cells to express various inflammatory cytokines [39, 40]. Among these inflammatory cytokines, tumor necrosis factor-alpha (TNF-*α*) decreased PS expression in rat SECs [30], and IL-6 increased PS expression and C4BP*β* expression, but not C4BP*α* expression, in rat hepatocytes [30, 31]. On the other hand, LPS directly decreased PS expression in human umbilical vein endothelial cells (HUVECs) (our unpublished observations). These findings are consistent with previous reports showing that TNF-*α* decreases PS expression in HUVECs [41] and that IL-6 increases PS expression in human hepatoma cell lines [34]. As described above plasma PS antigen and activity are decreased in LPS-treated rats, and this suggests that both LPS and TNF-*α* induce decreased plasma PS levels, which causes thrombotic tendency in patients with sepsis. On the other hand IL-6 increased PS expression in hepatocytes, but this IL-6-induced increase of PS expression is not enough to compensate for LPS- and TNF-*α*-induced decreased expression of PS in hepatocytes and/or endothelial cells. Furthermore, IL-6 itself increased C4BP expression in rat hepatocytes, and the resulting decrease of free PS in plasma is thought to be the cause of thrombotic tendency. Overall, these results suggest that various cytokines which are induced by LPS in monocytes and endothelial cells induced thrombotic tendency in patients with sepsis. Recently, it was reported that IL-6-induced increased PS expression in HepG2 cells is regulated through the signal transducer and activator of transcription (STAT3) binding site, which is located in the 5′-flanking region of the human PS gene [42]. The mechanisms by which TNF-*α* specifically decreases PS expression in SECs and IL-6 specifically increases C4BP*β* expression in hepatocytes are unknown. Further investigations are needed to elucidate the signal transduction pathway for IL-6 and TNF-*α* action in hepatocytes and SECs.

## 6. Conclusion

LPS directly decreases PS expression in hepatocytes and SECs and decreases C4BP expression in hepatocytes. Membrane-bound CD14 and TLR-4 mediate the LPS-induced activation of MEK/ERK and NF*κ*B and the activated NF*κ*B interacts with promoter regions of the PS and C4BP*α* and C4BP*β* genes. Moreover, LPS activates TLR-4 in monocytes to express IL-6 which stimulates expression of PS and C4BP in hepatocytes and/or endothelial cells. Our findings may be useful for the development of anticoagulation therapy involving PS and C4BP to regulate LPS-TLR-4-mediated activation of the NF*κ*B and MEK system.

## Abbreviations

LPS: Lipopolysaccharide
TLR: Toll-like receptor
PS: Protein S
C4BP: C4b-binding protein
IL-6: Interleukin-6
PKC: Protein kinase C
APC: Activated protein C
JNK: Jun kinase.
